# MicroRNAs in Brain Metastases: Potential Role as Diagnostics and Therapeutics

**DOI:** 10.3390/ijms150610508

**Published:** 2014-06-11

**Authors:** Samer Alsidawi, Ehsan Malek, James J. Driscoll

**Affiliations:** 1 Department of Internal Medicine, University of Cincinnati College of Medicine, Cincinnati, OH 45267, USA; E-Mails: alsidasr@ucmail.uc.edu (S.A.); maleken@ucmail.uc.edu (E.M.); 2 Division of Hematology and Oncology, University of Cincinnati College of Medicine, Cincinnati, OH 45267, USA; 3 The Vontz Center for Molecular Studies, University of Cincinnati College of Medicine, Cincinnati, OH 45267, USA

**Keywords:** brain metastases, miRNA replacement therapy, antagomirs

## Abstract

Brain metastases remain a daunting adversary that negatively impact patient survival. Metastatic brain tumors affect up to 45% of all cancer patients with systemic cancer and account for ~20% of all cancer-related deaths. A complex network of non-coding RNA molecules, microRNAs (miRNAs), regulate tumor metastasis. The brain micro-environment modulates metastatic tumor growth; however, defining the precise genetic events that promote metastasis in the brain niche represents an important, unresolved problem. Understanding these events will reveal disease-based targets and offer effective strategies to treat brain metastases. Effective therapeutic strategies based upon the biology of brain metastases represent an urgent, unmet need with immediate potential for clinical impact. Studies have demonstrated the ability of miRNAs to distinguish normal from cancerous cells, primary from secondary brain tumors, and correctly categorize metastatic brain tumor tissue of origin based solely on miRNA profiles. Interestingly, manipulation of miRNAs has proven effective in cancer treatment. With the promise of reduced toxicity, increased efficacy and individually directed personalized anti-cancer therapy, using miRNA in the treatment of metastatic brain tumors may prove very useful and improve patient outcome. In this review, we focus on the potential of miRNAs as diagnostic and therapeutic targets for the treatment of metastatic brain lesions.

## 1. Introduction

The treatment of metastatic brain tumors remains a daunting challenge. Metastatic brain lesions are the most frequently occurring intracranial tumors in adults with >170,000 patients diagnosed annually in the US—ten times the incidence of primary brain tumors [1,2]. Brain metastases continue to increase as a result of an aging population, the advent of targeted therapies that have increased the survival of patients with primary tumors and superior methods that allow earlier cancer detection [3]. The majority of brain metastases originate from primary lesions in the lungs (40%–50%), breasts (15%–25%) and melanomatous skin cancers (5%–20%) [4,5]. Median survival for patients with brain metastases is ~2 months if left untreated, but can be extended to 12–15 months with a multi-disciplinary approach, e.g., neurosurgery, radiosurgery and chemotherapy [6]. Irrespective of treatment, prognosis for patients with brain metastasis remains grim. The negative impact of metastatic brain tumors on patients extends beyond that of poor survival to include devastating effects on cognition, language, mobility and emotional well-being of patients and their families.

**Figure 1:**
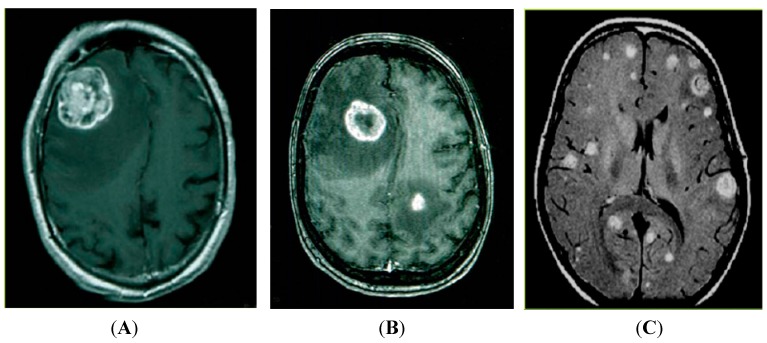
Magnetic resonance imaging (MRI) detection of brain metastasis. (**A**) Solitary lesion; (**B**) Oligometastasis; (**C**) Multiple brain metastases.

Lung cancer-derived brain metastases are an exceptionally important cause of morbidity and mortality since even small satellite lesions are incapacitating. Nearly 40% of lung cancer patients develop brain metastases during their disease lifetime [7]. At diagnosis, brain metastases can be detected in approximately 10% of all lung-cancer patients and in multiple retrospective series brain metastases are found in 50% of patients [8,9]. Magnetic resonance imaging (MRI) indicates that brain metastases can be detected as either solitary, oligometastases or as multiple lesions distinct from the originating primary tumor (Figure 1). Despite advances in the development of molecularly targeted therapies to treat primary lung tumors, most deaths from lung cancer result from the progressive growth of metastases that are resistant to current therapies.

**Figure 2:**
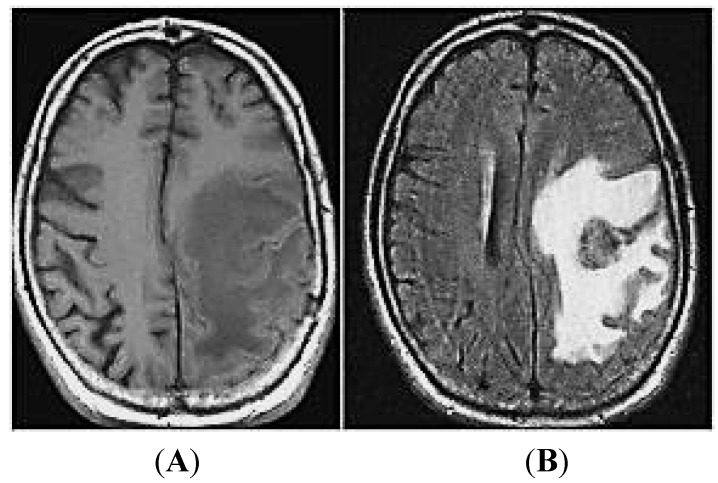
MRI of the brain to illustrate loss of blood–brain barrier integrity. Patient with metastatic brain lesion in the left cerebral hemisphere (**A**) before contrast; (**B**) after contrast. The leakage of contrast material (gadolinium) into and around the tumor rules out the blood–brain barrier as the sole mechanism for drug resistance.

Metastases develop when tumor cells successfully evade the homeostatic mechanisms within the host to exploit the cytoprotective features provided by the surrounding microenvironment. The “seed-and-soil” hypothesis of metastasis dictates that the successful outgrowth of deadly metastatic tumors depends on permissible, bidirectional interaction between the metastatic cancer cells and host tissue site-specific microenvironment [10]. However, the specific molecular networks, gene expression alterations and cellular signaling pathways needed to establish brain metastases remain poorly defined. Our understanding of the biology of brain metastases has improved dramatically in the last decade as a result of studies implementing animal models inoculated with high-level green fluorescent protein (GFP) labelled tumor cells and monitoring the formation of metastatic tumors *in vivo* using novel imaging techniques [11,12,13,14]. Current models of brain metastasis, such as transgenic and subcutaneous tumors implanted into immunodeficient mice, do not adequately represent the clinical scenario. Specifically, these models do not reflect the precise molecular steps involved in metastasis nor the response to therapeutic agents. To develop improved models, surgical orthotopic implantation (SOI) was developed to transplant histologically-intact human cancer cells or tissue, taken directly from patients, into the corresponding organ of immunodeficient mice. These unique SOI models have been successfully used for innovative drug discovery and mechanistic studies and serve as a bridge to link pre-clinical studies with clinical research and drug development. These highly valuable model systems should also be useful in validating miRNA therapeutics and complement imaging systems in the study of miRNA diagnostics and therapeutics. Histologic examination of tissue from human patient and animal models of brain metastases has revealed that these tumors are surrounded and infiltrated by reactivated astrocytes [15]. Astrocytes are the most common cell type in the brain and contribute to cerebral homeostasis through diverse methods [16]. Astrocytes support the blood–brain barrier (BBB), regulate blood flow, control inflammatory responses and participate in synaptic transmission. Astrocytes have also been shown to control extracellular homeostasis by regulating ion and glucose concentrations, acid-base balance and the supply of metabolites to neurons. Brain metastases surrounded by activated astrocytes are resistant to chemotherapy [15]. The metastatic tumor cells take advantage of the normal protective role of astrocytes which is to protect neural cells from toxins and exploit them to gain protection from chemotherapeutic agents. The brain was considered a sacred place and the resistance of metastatic tumor cells in the brain to chemotherapeutic drugs was falsely attributed to the inability to penetrate through the BBB, which is composed of endothelial cells with tight junctions enwrapped with basement membrane, pericytes and astrocytes. However, tumor cells within the brain parenchyma release vascular endothelial growth factor (VEGF) and other cytokines that increase vessel permeability [17,18]. Newer imaging techniques have proven that the BBB is dysfunctional in brain metastases as evidenced by leakage of contrast material into and around the tumors which basically rules out the BBB as the sole mechanism of drug resistance (Figure 2).

The formation of brain metastasis reflects the generalized process of cancer metastasis and consists of sequential, interlinked, and selective steps. The outcome of each step is influenced by the interaction of metastatic cells with homeostatic factors. Each step of the metastasis is considered rate limiting in that failure of a tumor cell to complete any step effectively terminates the process. Therefore, the formation of clinically relevant metastases represents the survival of unique subpopulations of cells that preexist in primary tumors. The successful formation of clinically significant metastatic tumor is thought to be the final product of survival specific cells within the primary tumor, *i.e.*, metastases-initiating cells. A key event of brain metastasis is the migration of cancer cells through the BBB. Although preventing brain metastasis is immensely important for survival, very little is known about the early stage of transmigration and the molecular mechanisms of tumor cells penetrating the BBB. The brain endothelium plays an important role in brain metastasis. Brain Microvascular Endothelial Cells (BMECs) are the major cellular constituent of the BBB and are joined by intercellular tight junctions responsible for maintaining selective permeability. BBB failure is critical in the development and progression of several diseases that affect the central nervous system (CNS), including brain tumor metastasis development. Crossing the BBB is rate limiting in the development of brain metastases. The presence of brain tumors disrupts the normal BBB, and it is now accepted that when a brain lesion grows beyond 1–2 mm the BBB becomes structurally and functionally compromised [19,20,21]. Over-expression of *p*-glycoprotein, a membrane protein that expels drugs from a cell’s cytoplasm, has also been implicated in chemoresistance [22,23]. Inhibiting *p*-glycoprotein, however, has not proved successful in reversing chemoresistance. Collectively, these studies indicate that unidentified mechanisms underlie the pro-survival effect of the brain microenvironment which has led to the search for genetic regulators.

## 2. MiRNAs and Brain Metastases

Genetic and epigenetic changes allow cancer cells to find the brain microenvironment—“the soil”—a favorable niche for tumorigenic “seeds” to implant, grow and blossom [24]. However, the precise manner in which the brain microenvironment promotes the growth of solid tumor cells remains a critical barrier. Understanding the precise micro-environment-mediated genetic events triggered in metastatic tumor cells to promote growth and drug resistance should substantially improve our knowledge base and identify new “druggable” targets. Non-coding (nc) RNAs are master regulators of the human genome and their aberrant expression contributes to tumorigenesis, metastasis and the acquisition of therapeutic resistance. However, the precise role of ncRNAs in brain metastases and the acquisition of drug resistance remained unknown. MiRNAs are endogenously expressed, small, non-coding RNA molecules that negatively regulate gene expression at the post-transcriptional level by base pairing to the 3' untranslated region (UTR) of target messenger RNA (mRNA). MiRNAs play a key role in cell development, proliferation, differentiation and apoptosis and, accordingly, alterations in miRNA expression are seen in tissues from all organ systems and contribute to cancers, autoimmune and genetic disorders and infectious processes. The loss of a tumor suppressive miRNA activates inherently oncogenic pathways to promote the generation of a cancer phenotype, tumor initiation, progression and metastasis [25]. Epigenetic alteration of miRNA have also been shown to play a role in cancer since compelling evidence demonstrates that miRNA deregulation promotes generation of a cancer phenotype, tumor initiation, metastatic growth and development of drug resistance [26,27]. Nearly 50% of human miRNA genes are located in areas of the genome associated with carcinogenesis [28]. Studying the miRNA profile of different tumors has gained popularity in the last decade as represents a breakthrough method for tumor classification that can impact cancer diagnosis, prognosis and treatment decisions. For example, miRNA profiling of 103 lymph node negative, breast cancer tumors lead to the identification of miRNA-106b in triple negative tumors and now known to carry a worse prognosis [29]. Different stages of breast cancer were noted to correlate with distinct miRNA profiles, including members of the miR-200 family and miR-9, to suggest that miRNAs are directly involved in tumor progression and metastasis. In colorectal cancer, the detection of circulating miRNA-141 correlated with metastatic disease and poor prognosis [30] and up-regulation of miR-9 was involved in metastases as well through facilitated cell motility and down-regulated α-catenin [31]. Certain miRNAs, e.g., miRNA-34 and let-7, were also found to be directly involved with the survival of tumor-initiating (or metastases-initiating) cancer stem cells (CSCs) [32,33]. MiRNA signatures have been identified within individual tumor types and may improve useful as diagnostics or prognostics of therapeutic response.

MiRNA profiling of tumor tissue may facilitate the identification of primary tumors based upon the miRNA profile of the metastatic brain lesion. Even with advanced imaging techniques, a small percentage of metastatic brain tumors remain of unknown origin. A recent study successfully identified the tumor of origin in 84% of brain metastases using a quantitative real-time polymerase chain reaction (qRT-PCR) of 48 different miRNAs [34]. Other studies have shown that miR-92b and miRNA-9/9* are over-expressed in primary brain tumors compared to metastatic brain tumors to aid in the diagnosis of brain lesions [35].

The role of miRNAs in the biology of brain metastases has been established in studies investigating a number of primary tumor types (Table 1) [36,37,38,39,40]. In breast cancer, miR-1258 alterations were directly related to heparanase expression, a known prometastatic enzyme found in brain metastatic breast cancer cells that degrades heparan sulfate chains to affect the cytoskeleton and render cells more capable of crossing the BBB [41,42]. The migratory and invasive capacity of breast CSCs was found to be related to the *KLF4* gene expression which is inversely related to miRNA-7 expression [43]. Similarly, in lung cancers, miRNA-145 down-regulation was involved in the growth of lung adenocarcinoma and promoted the formation of brain metastases [44]. MiRNA-328 in non-small cell lung cancer (NSCLC) regulated cell migration and the formation of brain metastases through altered expression of the *PRKCA* genes [45]. MiRNA-378 promoted brain metastases in NSCLC by increasing expression levels of MMP-7, MMP-9 and VEGF and decreasing levels of *Sufu*, all key genes involved in angiogenesis and extracellular matrix invasion [46]. MiRNA-200 family members were exclusively elevated in the CSF of patients with metastatic brain lesions from various primary tumor types when compared with glioblastoma and non-cancer patients [47].

**Table 1:** MiRNAs deregulated in brain metastases compared to the primary tumor. Deregulated miRNAs identified in metastatic brain tumor cells compared to their matched primary tumors. NSCLC, non-small cell lung cancer; MMP, matrix metalloproteinase; VEGF, Vascular endothelial growth factor; PTB1b, protein tyrosine phosphatase-1B; HIF-1α: Hypoxia-inducible factor 1-α.

Deregulated MiRNA	Direction of Expression in Brain Metastases	Primary Tumor	Putative Target
miR-1258 [41]	Down-regulated	Breast	Heparanase
miR-7 [43]	Down-regulated	Breast	*KLF4* gene
miR-145 [44]	Down-regulated	Lung adenocarcinoma	3'-UTR of the JAM-A and fascin
miR-146-a [48]	Down-regulated	Breast	B-catenin and hnRNPC
miR-768-3p [49]	Down-regulated	Lung and breast	K-RAS
miR-19a [50]	Down-regulated	Breast	3'-UTR of tissue factor transcript [36]
miR-29c [50]	Down-regulated	Breast and melanoma	Induced myeloid leukemia cell differentiation protein MCL1 [37]
miR-31 [51]	Down-regulated	Colon	p53 [38]
miR-328 [45]	Up-regulated	NSCLC	*PRKCA* gene
miR-378 [46]	Up-regulated	NSCLC	MMP-7, MMP-9 and VEGF
miR-200 [47]	Up-regulated	Breast and lung	E-cadherin transcriptional repressors ZEB1 and ZEB2 [39]
miR-210 [50]	Up-regulated	Breast and melanoma	PTP1b and HIF-1α [40]
miR-1, miR-145, miR-146a, miR-143, miR-10b, miR-22 [51]	Up-regulated	Colon	Multiple genes related to apoptosis and oncogenesis

**Figure 3:**
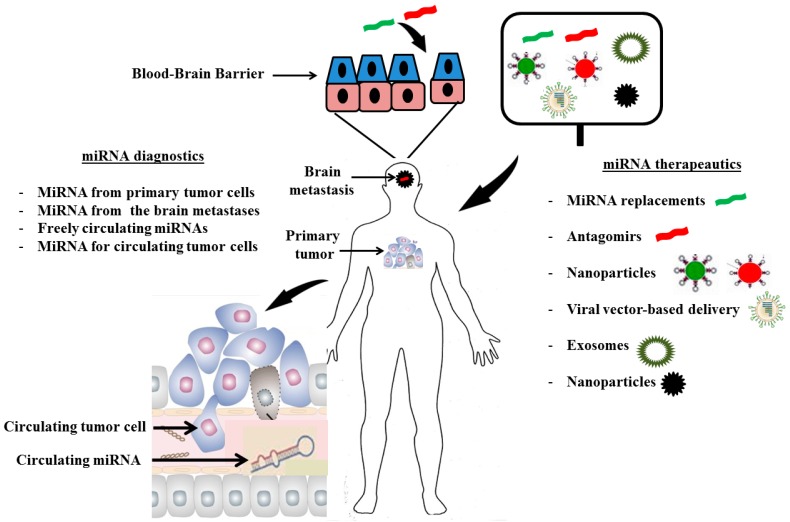
MiRNA diagnostics and therapeutics for brain metastases. A synthesized antisense nucleotide (antagomir, red) or miRNA replacement (green) is loaded onto a delivery system. The delivery system can be a viral vector such as adenovirus or a non-viral liposome or nanoparticle. The preparation is then administered intravenously to the patient with a metastatic brain tumor and remains stable in the blood stream. The compound crosses the blood–brain barrier and reaches the tumor cells and undergoes endocytosis to the intracellular space. The antagomir is then released from the delivery system which gets degraded. The antagomir binds to the miRNA of interest in blue and antagonizes its oncogenic effect which eventually leads to apoptosis and tumor regression.

The brain micro-environment, represented mainly by the astrocytes, is an active player and key regulator in the increased growth and chemoresistance of metastatic brain tumors (Figure 3). Astrocytes up-regulate a number of survival genes within the neighboring tumor cells and render these cells more aggressive, independent of primary tumor histology or *p*-glycoprotein activity [15]. MiRNAs are directly involved in the changes that the brain microenvironment implies on the metastatic tumor cells as many studies have shown that the brain microenvironment change the miRNA profile of the tumor cells when compared with the primary tumor. Rhabdoid tumor cells showed different miRNA profiles when originated in the brain compared to the kidney [52]. MiRNA-146a was noted to be suppressed in brain metastases compared to the original tumors in animal models, associated with decreased β-catenin protein levels and increased heterogeneous nuclear ribonucleoprotein C1/C2 (hnRNPC) which may increase migratory and invasive capabilities [48]. MiRNA-768-3p was down-regulated in tumor cells when co-cultured with astrocytes and this was validated in human brain metastatic tissues from lung cancer, breast cancer and melanoma when compared to match-paired primary tumor from the same patient. MiRNA-768-3p down-regulation led to an increase in *K-ras* expression and translated into increased tumor growth and drug resistance [49]. Different miRNA profiles were found between primary colorectal tumors and matched metastatic brain tumors [51] where over-expression of miRNA-145, 1, 146a, 576-5p, 126*, HS287, 28-5p, 143, 199b-5p, 199a-5p, 10b, 22, 133b, 145*, 199a, 133a, 125b and down-regulation of miRNA-31 and HS170 were observed in brain-metastatic carcinomas. Moreover, miRNAs isolated from exosomes of parental breast cancer and melanoma cells were different from those isolated from their corresponding metastatic brain variants. MiRNA-210 was over-expressed while miRNAs-19a and 29c were down-regulated in brain metastases [50]. These studies demonstrate that the brain microenvironment induces changes in the miRNA signature of the tumor cells to activate pro-growth signaling pathways and leads to more aggressive, drug resistant metastatic lesions. Studies suggest that the microenvironment influence on tumor cells that “seed” in the brain may be a universal effect [47]. This represents such an appealing concept to target key miRNAs involved in metastasis.

## 3. MiRNA Diagnostics

Understanding of the role of miRNAs in the biology of brain metastases has generated a greater demand to practically apply this knowledge in clinical practice. MiRNAs hold promise as diagnostics, prognostics and therapeutics to improve cancer patient outcome [53]. For example, miRNAs are being developed to improve detection of the plasma cell dyscrasia multiple myeloma (MM) [54]. Similarly, miRNA-based diagnostics may more readily detect metastatic brain lesions and distinguish primary from metastatic lesions [34]. MiRNA signatures may eventually be incorporated in clinical decision making as prognostic indicators to formulate treatment plans. Multiple miRNA signatures in primary tumors were shown to correlate with more aggressive, invasive, “brain-seeking” behavior. MiRNA-378 in NSCLC is associated with a greater likelihood of tumor seeding within the brain [46]. Clinical trials are needed to determine if miRNA signatures are predictive of worse prognosis. Such signatures could trigger more intensive treatment plans, e.g., prophylactic cranial irradiation or targeted therapy, to prevent the development of metastases or cranial recurrence. Early detection of brain micrometastases may be based upon deregulated miRNAs known to be altered within metastatic brain tumors. Changes in miRNA levels, e.g., loss of miRNA-768-3p signal [49] or increase in miRNA-200 [47], may provide an early signal to prompt aggressive treatment. MiRNAs are also readily detectable and stable within human plasma [30,55,56]. These miRNAs are protected from endogenous RNase activity as free-circulating molecules, within circulating tumor cells (CTCs) or in membrane-derived small membrane vesicles, exosomes, that are released by cells [57]. MiRNAs from plasma, CTCs and exosomes have been successfully detected using RT-PCR techniques and may serve as readily-available diagnsotics [58,59,60]. Deregulated levels of miRNAs have been detected in the plasma of patients with lymphoma (miRNA-155, 210, 21) [61], leukemia (miRNA-92, 150, 342) [62,63], colon cancer (miRNA-29a, 92a) [64], breast cancer (miRNA-195, 21, 92a, let-7a) [65,66], prostate cancer (miRNA-375, 141) [67], ovarian cancer (miRNA-21, 92, 93) [68], pancreatic cancer (miRNA-155, 196a, 642b, 885, 5p, 22, 16) [69,70,71,72], gastric cancer (miRNA-17, 1, 106a, 106b, let-7a and 18a) [73,74,75] and lung cancer (miRNA-486, 30d, 1, 499 and 375) [58,76,77]. MiRNAs were also found to be stable in the cerebrospinal fluid (CSF) of patients with neoplasms as well as neurologic disorders [47,78]. Given the relative invasive nature of CSF sampling, the challenge in miRNA diagnostics in brain metastases is the BBB and whether miRNAs (either as free molecules, in CTCs or other form of transport system such as exosomes) are able to cross the BBB and be readily detectable in the serum of patients. Studies in glioblastomas have shown that miRNA signals can be detected within exosomes in the serum of these patients [59]. These results support disruption of the BBB during metastasis [19]. Studies also detected relevant miRNAs in the plasma of Alzheimer’s or Huntington’s disease patients even though the BBB is thought to remain intact in these conditions [79]. Membrane-derived extracellular vesicles (EVs) containing miRNAs originate from CNS tumors and may function as intercellular communication with the microenvironment and across the BBB [80]. Now that it has been established that the miRNA profile of brain metastases is distinct from primary tumors, it would be of great importance to be able to routinely and inexpensively detect these miRNAs in the blood, serum, CSF or urine of patients.

## 4. MiRNA Therapeutics

Discoveries in miRNA biology, and their close relationship to oncogenesis in many tumor types, has led to attempts to translate this information into miRNA therapeutics (Figure 3) [81,82]. Currently, however, there is a lack of miRNA-based therapeutics to directly target brain metastases. A well-established feature is that a single miRNA is capable of regulating multiple genes, which makes endogenous miRNAs appealing therapeutic targets. Altering miRNA signatures was also found to sensitize tumor cells to other forms of treatment in, otherwise, chemo-resistant tumors [83]. Two strategies exist for miRNA-based therapeutics: a direct approach which involves either miRNA mimics to replace the loss of a tumor suppressor miRNA or miRNA antagomirs which are antisense oligonucleotides that block oncogenic miRNAs; and an indirect strategy that involves identifying existing agents that modulate the expression and/or processing of miRNAs in traditional compound-library screens.

**Figure 4:**
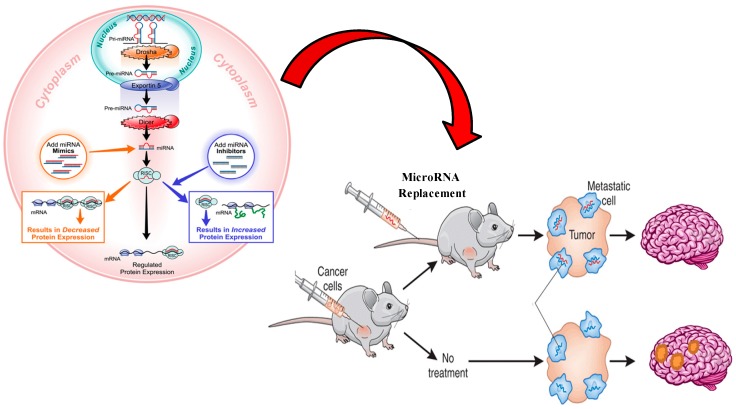
Model to illustrate the effect of microRNA-based therapeutics for the treatment of brain metastases at the cellular and animal levels. Oncology-directed miRNA replacement therapy. Loss of a tumor suppressor miRNA leads to hyperactivation of inherently oncogenic pathways and tumorigenesis. Administration of a miRNA mimic reinstates the function of the missing tumor suppressor miRNA, suppresses oncogenic pathways and cancer cell growth.

The development of antagomirs went through multiple phases to increase their stability since naked RNA has a very short half-life in the bloodstream and the use of phosphodiester oligodeoxynucleotides (ODNs), without further modification was unsuccessful [84]. The *in vivo* stability of antagomirs has been augmented by multiple chemical modifications such as the development of phosphorothioate containing oligonucleotides [85], 2'-*O*-methyl-(2'-*O*-Me) or 2'-*O*-methoxyethyl-oligonucleotides (2'-*O*-MOE) which improves ribonuclease resistance and increases the binding affinity to the miRNA [86], locked nucleic acid (LNA) oligonucleotides where the ribose ring is “locked” by a methylene bridge which further increases the affinity towards single stranded RNAs [87,88], peptide nucleic acids (PNA) which are artificially synthesized polymers similar to RNAs but are resistant to enzyme degradation [89] and fluorine-derivative nucleic acids (FANA and 2'-F) [90]. Similar to antagomirs, miRNA sponges inhibit miRNA where plasmids containing multiple tandem-binding sites to the miRNA of interest are transfected into the cells and help “fool” the miRNA into binding to the sponge instead of its target mRNA [91]. MiRNA masks are single-stranded 2-*O*-methyl antisense oligonucleotides that are complementary to the supposed miRNA binding sites in the 3'-UTR of the mRNA [92]. MiRNA replacement therapy aims at restoring a tumor suppressor miRNA that is down-regulated in tumor cells with oligonucleotide mimics similar to the original miRNA. Using longer strands that mimic the pre-miRNA have also been proposes but these require different delivery systems to ensure intranuclear availability [93]. The delivery of miRNA mimics with tumor suppressor effect into tumor cells have shown to be effective in inducing cell death (Figure 4) [49,94,95,96].

A current dilemma in miRNA therapeutics is an efficient system to guarantee stability in the blood and adequate delivery to tissues of interest. Viral and non-viral delivery methods have been used with variable success. Adenovirus-associated vectors (AAV) emerged as an appealing method since they have acceptable toxicity profiles [97] and were successfully injected intravenously in mouse models to restore miRNA-26 expression in hepatocellular carcinoma cells [98]. Different AAV serotypes can successfully target distinct tumor types. Non-viral delivery methods may be superior to AAV methods given their stable formulations. For example, liposomes composed of phospholipid bilayers were used to deliver miRNA-133b to lung cancer cells in mice [99]. Liposome use, however, is limited by their toxicity, related to their strong cationic charge [100]. Liposomes have gone through multiple levels of development to improve their stability and minimize the side effects. Hyaluronic acid was added to form polycationic liposome-hyaluronic acid (LPH) which successfully delivered siRNA and miRNA-34a into mouse melanoma models [101]. To overcome the toxicity of liposomes, a neutral lipid emulsion was developed which has a natural predilection to accumulate in the lung compared to the liver predilection of cationic liposomes. The neutral lipid emulsion successfully delivered let-7 and miRNA-34a to lung cancer cells in mice [102]. Liposomes have a short half-life and require continuous infusion or frequent administration which limits their use. Multiple attempts were made to overcome these problems which led to the development of sustained-release polymer formulations [103]. Other forms of non-viral delivery systems include dendrimers which are repetitively-branched perfectly-structured particles that have a high surface to volume ratio and were successfully used in delivering anti-miR-21 and 5-flurouracil to glioblastoma cells *in vitro* [104]. Other nanoparticles, microspheres and hydrogels have been developed [105] such as the polylactide-*co*-glycolide (PLGA) particles which are stable particles that allow the delivery of miRNA over time and are highly adaptable and can be used to load multiple cargos. PLGA particles delivered anti-miRNA-155 to malignant pre-B lymphoma cells in mouse models with good results [106].

Throughout the different stages of development of gene and miRNA delivery systems, a major obstacle has been crossing the BBB. Although surgically-implanted wafers and intra-thecal routes are established methods to administer chemotherapy, oral or intravenous routes remain the most convenient. The BBB only allows lipophilic molecules, less than 400 Da, to penetrate the CNS [107]. A few novel techniques have been used to overcome this obstacle. The Trojan Horse Liposome (THL) system encapsulates the genomic material, *i.e.*, miRNA replacements or antagomirs, within the liposome to protect it from nuclease degradation. The compound is constructed using polyethyleneglycol (PEG) to stabilize the liposome [108]. Part of the PEG can be engineered with peptidomimetic monoclonal antibodies (mAbs) that target specific BBB receptors (such as the insulin receptor or the transferrin receptor) and facilitate the transcytosis of the compound. The THL technology has been used to administer compounds that cross the BBB and deliver genetic material to the CNS [109]. Another novel method to bypass the BBB is through polyethylenimine (PEI)-based delivery systems as are widely used in gene therapy [110]. PEI complexes are positively charged that bind negatively charged nucleic acid, *i.e.*, miRNAs. The compound retains an overall positive charge that interacts with negatively charged polysaccharides on the cell surface. This process is followed by endocytosis of the compound to evade the endosome by inducing an influx of protons and water leading to swelling and disruption of the endosome and release of the compound containing the miRNA in the cytoplasm. PEI-based systems have been modified to cross the BBB by adding a short peptide inspired from the rabies virus glycoprotein (RVG) which binds the acetylcholine receptor [111]. Mannitol also is added to increase the permeability of the BBB [112]. The PEI–RVG compound crossed the BBB and delivered the neuron specific miR-124a to brain cells. Even with rapidly emerging understanding of miRNA biology and the development of novel delivery systems, the clinical use of miRNA therapeutics to treat brain metastases remains limited in pre-clinical development and has yet to be exploited. The previous misconception of the brain as a sanctuary organ that systemic or targeted therapies cannot penetrate has contributed to delays in clinical advancement. Future studies are needed to better define miRNA signatures within brain metastases and to correlate these signatures with the miRNA profile of the primary tumor. Advances have been made in pre-clinical and translational studies to identify miRNAs that change after growth in the brain microenvironment but require validation from patient tumor samples [49].

## 5. Conclusions

Despite advances in developing miRNA diagnostics and therapeutics, significant challenges remain. Since miRNA are upstream regulators of hundreds of genes, the off-target effects of miRNA therapeutics are a potential limitation. Toxicities associated with miRNA therapeutics are not limited to the delivery system since studies have shown that oversaturating small RNA pathways can be lethal [113]. The induction of interferon-α through the toll like receptor (TLR-7) by short interfering RNA (siRNA) leads to systemic immune responses and poor outcomes [111]. The availability of a reliable delivery system that has minimal toxicities, crosses the BBB and successfully unloads the miRNA therapeutic is needed to promote clinical advancement (Figure 4).

In summary, the survival of patients with brain metastases remains poor due to the lack of effective treatments. MiRNAs are key regulators of gene expression and their role in multiple cancer types is well-established. Multiple miRNA signatures are altered in brain metastases relative to the primary tumor and are, in fact, induced through interaction with the brain microenvironment. Identifying miRNA signatures within brain metastases represents a promising approach to target these lesions. However, numerous challenges exist in translating this information into clinical practice. MiRNA therapeutics may eventually provide individualized therapy for patient and this approach is applicable to molecularly heterogeneous diseases with distinct genetic subtypes, such as brain metastases [54].
